# Preliminary results of the use of urinary excretion of pyridinium crosslinks for monitoring metastatic bone disease.

**DOI:** 10.1038/bjc.1992.161

**Published:** 1992-05

**Authors:** R. E. Coleman, S. Houston, I. James, A. Rodger, R. D. Rubens, R. C. Leonard, J. Ford

**Affiliations:** Department of Clinical Oncology, Western General Hospital, Edinburgh, UK.

## Abstract

The collagen crosslinks, pyridinoline and deoxypyridinoline, are recently described markers of the rate of bone resorption. The urinary excretion of these compounds, expressed as a ratio to urinary creatinine, has been measured using ion-pair reversed phase high-performance liquid chromatography in 20 patients receiving oral pamidronate for bone metastases from breast cancer. Before treatment the ratio of pyridinoline and deoxypyridinoline to creatinine in urine (UPCR and UdPCR respectively) were each above normal in 16/20 (80%) patients. Urinary calcium excretion (UCCR) was elevated in 15/20 (75%). There was a strong correlation between UPCR and UdPCR, but neither of the crosslink measurements correlated well with UCCR. Urinary excretion of all three indices of bone resorption fell significantly during pamidronate treatment. The median values after 4 weeks treatment were 63% of baseline for UPCR, 45% for UdPCR and 26% for UCCR. From this preliminary study urinary pyridinoline and deoxypyridinoline excretion appear to be promising markers of bone resorption in advanced malignancy. Their role in response assessment and the advantages over UCCR measurements merit further study.


*Br. J. Cancer* (1992), **65**, 766–768 © Macmillan Press Ltd., 1992

### Preliminary results of the use of urinary excretion of pyridinium crosslinks for monitoring metastatic bone disease

R.E. Coleman[1], S. Houston[2], I. James[3], A. Rodger[1], R.D. Rubens[2], R.C.F. Leonard[1] & J. Ford[4]

[1]*Department of Clinical Oncology, Western General Hospital, Edinburgh;* [2]*ICRF Clinical Oncology Unit, Guy's Hospital, London;* [3]*Department of Metabolic Medicine, London Hospital, London, UK;* [4]*Ciba-Geigy Ltd, Basle, Switzerland.*

**Summary** The collagen crosslinks, pyridinoline and deoxypyridinoline, are recently described markers of the rate of bone resorption. The urinary excretion of these compounds, expressed as a ratio to urinary creatinine, has been measured using ion-pair reversed phase high-performance liquid chromatography in 20 patients receiving oral pamidronate for bone metastases from breast cancer.

Before treatment the ratio of pyridinoline and deoxypyridinoline to creatinine in urine (UPCR and UdPCR respectively) were each above normal in 16/20 (80%) patients. Urinary calcium excretion (UCCR) was elevated in 15/20 (75%). There was a strong correlation between UPCR and UdPCR, but neither of the crosslink measurements correlated well with UCCR. Urinary excretion of all three indices of bone resorption fell significantly during pamidronate treatment. The median values after 4 weeks treatment were 63% of baseline for UPCR, 45% for UdPCR and 26% for UCCR.

From this preliminary study urinary pyridinoline and deoxypyridinoline excretion appear to be promising markers of bone resorption in advanced malignancy. Their role in response assessment and the advantages over UCCR measurements merit further study.

Breast cancer commonly causes destructive lytic bone metastases characterised by an accelerated rate of bone resorption. Several biochemical markers of bone metabolism have been studied as indicators of bone response (Hortobagyi *et al.*, 1984; Coleman *et al.*, 1988*a*). These have included urinary calcium (Campbell *et al.*, 1983) and hydroxyproline excretion (Blomqvist *et al.*, 1987) which are influenced by the rate of bone resorption. However, they have significant limitations for routine use in response assessment. Firstly, urinary calcium excretion reflects the net difference in the rate of bone resorption and formation. Although typically increased in breast cancer, it may be normal, and therefore probably unhelpful, in patients with predominantly sclerotic metastatic bone disease. Secondly, hydroxyproline excretion is not specific for bone collagen resorption and profoundly influenced by diet, collagen synthesis, complement activation and particularly in malignancy, soft tissue destruction by extraskeletal metastases. Levels may therefore reflect changes in these confounding factors rather than changes in bone metabolism.

Recently, a new class of potential markers of bone turnover have been identified (Eyre & Oguchi, 1980; Beardsworth *et al.*, 1990). These are the crosslinking amino acids of collagen, pyridinoline (also known as hydroxylysylpyridinoline), and deoxypyridinoline (also known as lysylpyridinoline). The urinary excretion of pyridinoline and deoxypyridinoline can now be reliably measured (James *et al.*, 1990) and are specific measures of the rate of bone resorption (Uebelhart *et al.*, 1990).

Elevated levels of pyridinoline and deoxypyridinoline compared with normal subjects have been reported in metabolic bone disease (Uebelhart *et al.*, 1990 and 1991) and in a small series of patients with bone metastases (Paterson *et al.*, 1991). However, to our knowledge there have been no reports of the effects of treatment for bone metastases on cross-link excretion. In this study we have measured pyridinoline and deoxypyridinoline in patients with bone metastases from breast cancer before and during treatment with oral pamidronate.

Correspondence: R.E. Coleman, Senior Lecturer and Honorary Consultant, Department of Clinical Oncology, Weston Park Hospital, Whitham Road, Sheffield S10 2SJ, UK.

Received 28 June 1991; and in revised form 3 January 1992.

### Methods

Twenty women with bone metastases from breast cancer were recruited as part of a multicentre tolerability and efficacy study of a new enteric-coated micropellet of pamidronate developed by Ciba Geigy Ltd, Basle. Patients had either radiographic evidence of progressive bone disease, or apparently stable disease for at least 6 months, but with symptoms which justified a treatment change. Patients were randomly allocated to receive oral pamidronate at one of three dose levels, 75 mg daily ($n = 7$), 150 mg daily ($n = 5$) or 300 mg daily ($n = 8$) for 4 weeks. Patients received no other systemic therapy for breast cancer except for those already on endocrine treatment who continued with this to prevent the confounding effect of a withdrawal response. After 4 weeks the study period ended and pamidronate was continued with or without other systemic treatments as appropriate. For the purposes of this study the data are confined to the 4 week study period.

Patients were evaluated before treatment and at weekly intervals for 4 weeks during treatment. Blood was taken at each visit for routine haematology and biochemistry and the tolerability of oral pamidronate recorded (to be published elsewhere). On the morning of each assessment, patients collected the second early morning fasting sample of urine voided. The urine was acidified with 1.5 ml of normal (3%) hydrochloric acid for every 20 ml of urine and stored at −20° for subsequent measurement of pyridinoline, deoxypyridinoline, calcium and creatinine.

The HPLC method for measurements of pyridinoline and deoxypyridinoline has been described in detail elsewhere (James *et al.*, 1990). Briefly, the assay used ion-pair reversed-phase high performance liquid chromatography (HPLC) in the presence of 1-octanesulphonic acid (OSA). Pyridinoline and deoxypyridinoline were separated on a 5 μm ODS techsphere column (100 mm × 4.6 mm I.D.) [HPLC Technologies, Macclesfield]. The column was eluted with mobile phase consisting of 25 mM sodium formate, 5 mM OSA and 1 mM ethylenediaminetetraacetic acid (EDTA) adjusted to pH 3.25, containing 17.5% (v/v) methanol at 1.25 ml $min^{-1}$. Compounds were detected by their natural fluorescence (xenon lamp; excitation wavelength 290 nm, emission wavelength 400 nm) using a Jasco 821SP fluorimeter. The intra-assay coefficients of variation were 7.65% for pyridinoline and 9.07% for deoxypyridinoline. The limit of detection was 200 fmol. Sample results were expressed as a ratio of pyridinoline or deoxypyridinoline (in nmols $l^{-1}$) to creatinine

(in mmols $l^{-1}$) (UPCR and UdPCR respectively). The upper limits of normal in adult women for UPCR and UdPCR vary with age but using this technique were <30 and <10 (unpublished data). These are slightly lower than reported measurements on fasting early morning samples from post-menopausal women (Uebelhart *et al.*, 1991) but in accordance with the previously published normal ranges for measurements of UPCR and UdPCR performed on 24 h urine collections in post-menopausal women (Uebelhart *et al.*, 1990) and women aged 30–70 (Beardsworth *et al.*, 1990).

Urinary calcium ($\mu$mol $l^{-1}$) was measured using the cresolphthalein-complexone method and a colourimetric assay (Ciba-Corning Diagnostics, USA). Urinary creatinine (mmol $l^{-1}$) was measured using an enzymatic determination with creatininase/creatikinase (WAKO chemicals, Japan). The urinary calcium and creatinine were then expressed as a ratio (Peacock *et al.*, 1969). The upper limit of normal for the urinary calcium/creatinine ratio was taken as 300.

Standard statistical tests for calculation of correlation coefficients and *P* values were used. The paired students *t*-test was used to study differences between initial values of UCCR, UPCR and UdPCR and those during treatment (Armitage, 1971).

### Results

Pretreatment levels of UPCR and UdPCR were each elevated above the normal range in 16/20 (80%) of patients. The range of values for UPCR was 16–401 (median 71) and for UdPCR 2.1–95 (median 15). The UCCR was elevated in 15/20 (75%) with a range of values of 40–1,390 (median 600) (Figure 1). Only one patient had normal values of all three markers of bone resorption. The correlation between UPCR and UdPCR was strong ($r = 0.96$, $P = <0.001$) but neither of the crosslinks showed a significant correlation with UCCR ($r = 0.29$, $P = 0.5$ for UPCR, $r = 0.20$, $P = >0.5$ for UdPCR).

During treatment with pamidronate, as bone resorption was inhibited, urinary excretion of both collagen crosslinks and calcium reduced. After 4 weeks treatment the median values (expressed as a percentage of the pretreatment level) were 63% for UPCR, 45% for UdPCR and 26% for UCCR. Patients with elevated pretreatment levels showed significant falls in UCCR ($P = <0.001$) in 14/15 (93%), UPCR ($P = <0.01$) in 13/16 (81%) and UdPCR ($P = <0.001$) in 14/16 (88%) patients respectively (Figure 2). Changes in the few patients with normal baseline values were variable and difficult to interpret. The two patients who showed an increase in UPCR and UdPCR during treatment had a fall in UCCR. Both UPCR and UdPCR were normal in the patient who showed a rise in UCCR. A wide range of pre and post treatment values were seen, but based on the limited statistical power of such small groups, the reduction in pyridinoline crosslink excretion did not appear to be related to the daily dose of pamidronate.

**Figure 1** Urinary excretion of calcium in $\mu$mol l (UCCR), pyridinoline in nmol $l^{-1}$ (UPCR) and deoxypyridinoline in nmol $l^{-1}$ (UdPCR) before starting pamidronate ($n = 20$). Expressed as a ratio to creatinine excretion (mmol $l^{-1}$). The upper limit of normal for the ratios are as indicated.

**Figure 2** Changes after 4 weeks treatment with oral pamidronate in UCCR (top, $n = 15$), UPCR (middle, $n = 16$) and UdPCR (bottom, $n = 16$) in patients with initially elevated values. Values expressed as a percentage of baseline.

### Discussion

In recent years there has been a considerable interest in metastatic bone disease. Our understanding of the pathophysiological processes involved and the emergence of new treatments has prompted a search for reliable, reproducible and convenient markers of response. These are needed to complement plain radiography which can be difficult to interpret and which show structural disturbances that only slowly and unreliably reflect changes in the metastases and their effects on bone metabolism. Biochemical monitoring can predict response to treatment (Blomqvist *et al.*, 1987; Coleman *et al.*, 1988), but more specific markers of bone resorption and formation should improve on the results reported so far.

Studies of collagen structure have revealed that the collagen matrix is held together by intermolecular non-reducible crosslinking amino acids. In hard connective tissues such as bone, dentine and cartilage the predominant crosslinks are the naturally fluorescent pyridinium compounds pyridinoline, and deoxypyridinoline (Eyre & Oguchi, 1980). Pyridinoline is the major component in all these tissues while deoxypyridinoline is largely confined to bone and dentine where it comprises 21–22% of collagen crosslinks.

Both pyridinoline and deoxypyridinoline are almost completely excreted during collagen degradation (Segrest, 1982), are unaffected by diet, and found in free and conjugated forms in urine at levels of approximately 1 per 15,000 amino acid residues. Several methods have been developed for quantification of pyridinoline including cation-exchange chromatography (Fujimoto *et al.*, 1983), an enzyme-linked immuno absorbent assay (ELISA) directed against pyridinoline and deoxypyridinoline which can therefore not be measured separately by this technique (Robins, 1982), reverse phase HPLC (Black *et al.*, 1988) and, as used in this study a modified HPLC technique allowing rapid assay of both pyridinoline and deoxypyridinoline (James *et al.*, 1990).

Elevated levels of UPCR and UdPCR compared with normal subjects have been reported in Paget's disease of bone, hyperparathyroidism and osteoporosis. The raised levels reflect the increased rate of bone resorption associated with these conditions. Levels of UPCR and UdPCR then fall as resorption is inhibited following treatment with for example, pamidronate for Paget's disease (Uebelhart *et al.*, 1990) or hormone replacement therapy for osteoporosis (Uebelhart *et al.*, 1991). In this study the UPCR and UdPCR were elevated in 80% of our patients with metastatic bone disease. These results are similar to the findings of Paterson *et al.* (1991) who observed increased crosslinks excretion in eight out of ten patients with bone metastases.

Pamidronate is a potent inhibitor of osteoclast activity and the treatment of choice for hypercalcaemia of malignancy (Ralston *et al.*, 1989). Long-term administration of pamidronate has consistently shown a reduction in morbidity from bone metastases (van Holten-Verzantvoort *et al.*, 1987) and, in some patients, healing of lytic bone metastases has been observed (Coleman *et al.*, 1988*b*; Dodwell & Howell, 1991). In this study, testing a new oral formulation of pamidronate, the rate of bone resorption was increased in the majority of patients before treatment and then slowed, with a few exceptions, during treatment. UPCR and UdPCR did not correlate well with calcium excretion suggesting they reflect different facets of bone destruction. In addition, the fall in UPCR and UdPCR excretion during oral pamidronate treatment although significant, was of a smaller magnitude than the suppression of calcium excretion.

Previous trials with intravenous pamidronate have shown an initial fall in urinary calcium excretion in all patients. However, in some patients the calcium excretion rises again after the initial fall and, only a subset of patients achieving a normal calcium excretion which is maintained over several months who appear to benefit either symptomatically or radiologically (Coleman *et al.*, 1988*b*; Dodwell *et al.*, 1990). The explanations for both the unmaintained effect of pamidronate and a lack of response in some patients despite apparent inhibition of bone resorption are not known. Further studies are planned which will incorporate measurements of UPCR and UdPCR in the hope that these more specific markers of bone resorption will identify those patients who will benefit most from bisphosphonate treatment.

In conclusion, our preliminary results indicate that urinary excretion of pyridinoline and deoxypyridinoline is raised in patients with bone metastases and falls as bone resorption is inhibited by the bisphosphonate pamidronate. The value of these markers in a larger cohort of patients compared with an age-matched control population and their role in assessment of response in bone to endocrine and cytotoxic treatments is now being studied.

### References

ARMITAGE, P. (1971). *Statistical Methods in Medical Research.* Blackwell Scientific Publications: Oxford.

BEARDSWORTH, L.J., EYRE, D.R. & DICKSON, I.R. (1990). Changes with age in the urinary excretion of lysyl- and hydroxylysylpyridinoline, two new markers of bone collagen turnover. *J. Bone & Min. Res.*, **5**, 671.

BLACK, D., DUNCAN, A. & ROBINS, S.P. (1988). Quantitative analysis of the pyridinium crosslinks of collagen in urine using ion-pair reversed-phase high-performance liquid chromatography. *Anal. Biochem.*, **169**, 197.

BLOMQVIST, C., ELOMAA, I. & VIRKKUNEN, P. & 4 others (1987). The response evaluation of bone metastases in breast carcinoma. *Cancer*, **60**, 2907.

CAMPBELL, F.C., BLAMEY, R.W., WOOLFSON, A.M.J., ELSTON, C.W. & HOSKING, D.T. (1983). Calcium excretion $Ca_E$ in metastatic breast cancer. *Br. J. Surg.*, **70**, 202.

COLEMAN, R.E., WHITAKER, K.B., MOSS, D.W. & 3 others (1988*a*). Biochemical prediction of response of bone metastases to treatment. *Br. J. Cancer*, **55**, 61.

COLEMAN, R.E., WOLL, P.J., MILES, M., SCRIVENER, W. & RUBENS, R.D. (1988*b*). 3-amino-1,1 hydroxypropylidene bisphosphonate (APD) for the treatment of bone metastases from breast cancer. *Br. J. Cancer*, **58**, 621.

COOMBES, R.C., DADY, P., PARSONS, C. & 4 others (1983). Assessment of response of bone metastases to systemic treatment in patients with breast cancer. *Cancer*, **52**, 610.

DODWELL, D.J. & HOWELL, A. (1991). The systemic treatment of bone metastases. In: *Bone Metastases – Diagnosis and Treatment*, Rubens, R.D. & Fogelman, I. (eds) p. 121. Springer-Verlag: London.

DODWELL, D.J., HOWELL, A., MORTON, A., DALEY-YATES, P.T. & HOGARTH, C.R. (1990). Pamidronate (APD) treatment of skeletal metastases from breast cancer. In *The Management of Bone Metastases and Hypercalcaemia by Osteoclast Inhibition*, Rubens, R.D. (ed.) p. 62. Hogrefe & Huber Publishers: Bern.

EYRE, D.R. & OGUCHI, H. (1980). The hydroxypyridinium crosslinks of skeletal collagens: their measurement, properties and a proposed pathway of formation. *Biochem. Biophys. Res. Commun.*, **92**, 403.

FUJIMOTO, D., SUZUKI, M., UCHIMAYA, A., MIYAMOTO, S. & INOUE, T. (1983). Analysis of pyridinoline, a crosslinking compound of collagen fibers, in human urine. *J. Biochem.*, **94**, 1133.

HORTOBAGYI, G.N., LIBSHITZ, H.I. & SEABOLD, J.E. (1984). Osseus metastases of breast cancer. Clinical, biochemical, radiographic and scintigraphic evaluation of response to therapy. *Cancer*, **55**, 577.

JAMES, I.T., PERRETT, D. & THOMPSON, P.W. (1990). Rapid assay for hard tissue collagen cross-links using isocratic ion-pair reversed-phase liquid chromatography. *J. Chromatogr.*, **525**, 43.

PATERSON, C.R., ROBINS, S.P., HOROBIN, J.M., PREECE, P.E. & CUSCHIERI, A. (1991). Pyridinium crosslinks as markers of bone resorption in patients with breast cancer. *Br. J. Cancer*, **64**, 884–886.

PEACOCK, M., ROBERTSON, W.D. & NORDIN, B.E.C. (1969). Relation between serum and urinary calcium with particular reference to parathyroid activities. *Lancet*, **i**, 384.

RALSTON, S.H., GALLACHER, S.J., PATEL, U. & 3 others (1989). Comparison of three intravenous bisphosphonates in cancer-associated hypercalcaemia. *Lancet*, **ii**, 1180.

ROBINS, S.P. (1982). An enzyme-linked immunoassay for the collagen crosslink pyridinoline. *Biochem. J.*, **207**, 617.

SEGREST, J.P. (1982). Urinary metabolites of collagen. *Methods Enzymol.*, **82**, 398.

UEBELHART, D., GINEYTS, E., CHAPUY, M.-C. & DELLIAS, P.D. (1990). Urinary excretion of pyridinium crosslinks: a new marker of bone resorption in metabolic bone disease. *Bone & Mineral*, **8**, 87.

UEBELHART, D., SCHLEMMER, A., JOHANSEN, J. & 3 others (1991). Effect of menopause and hormone replacement therapy on the urinary excretion of pyridinium cross-links. *J. Clin. Endocrinol. & Metab.*, **72**, 367.

VAN HOLTEN-VERZANTVOORT, A.T., BIJVOET, O.L.M. & HERMANS, J. & 8 others (1987). Reduced morbidity from skeletal metastases in breast cancer patients during long-term bisphosphonate (APD) treatment. *Lancet*, **ii**, 983.


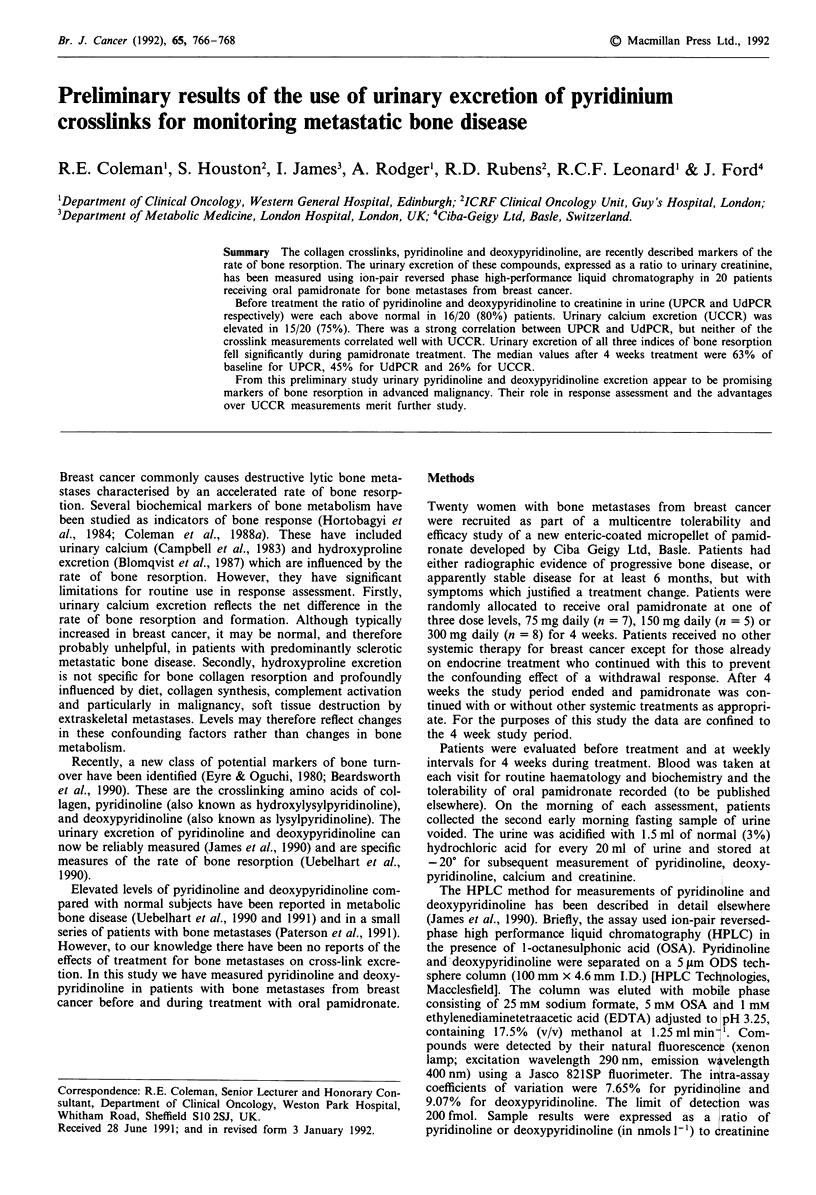

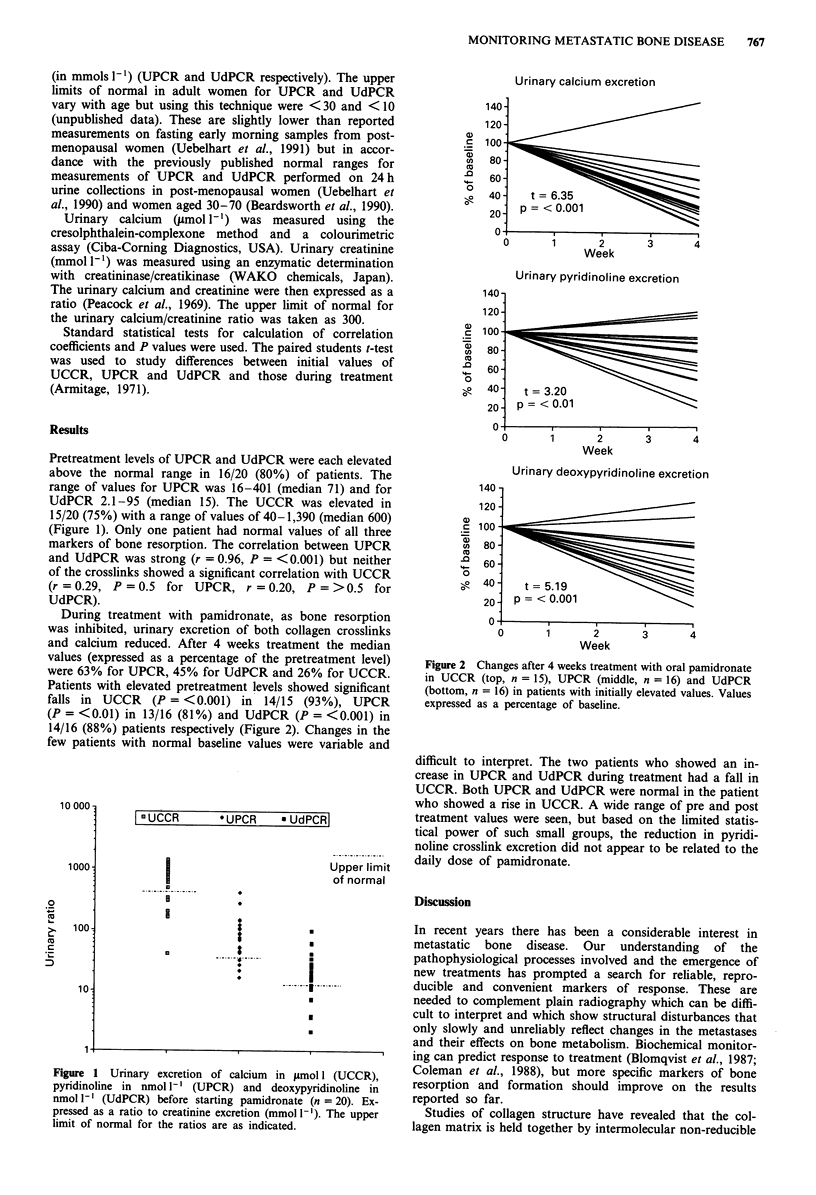

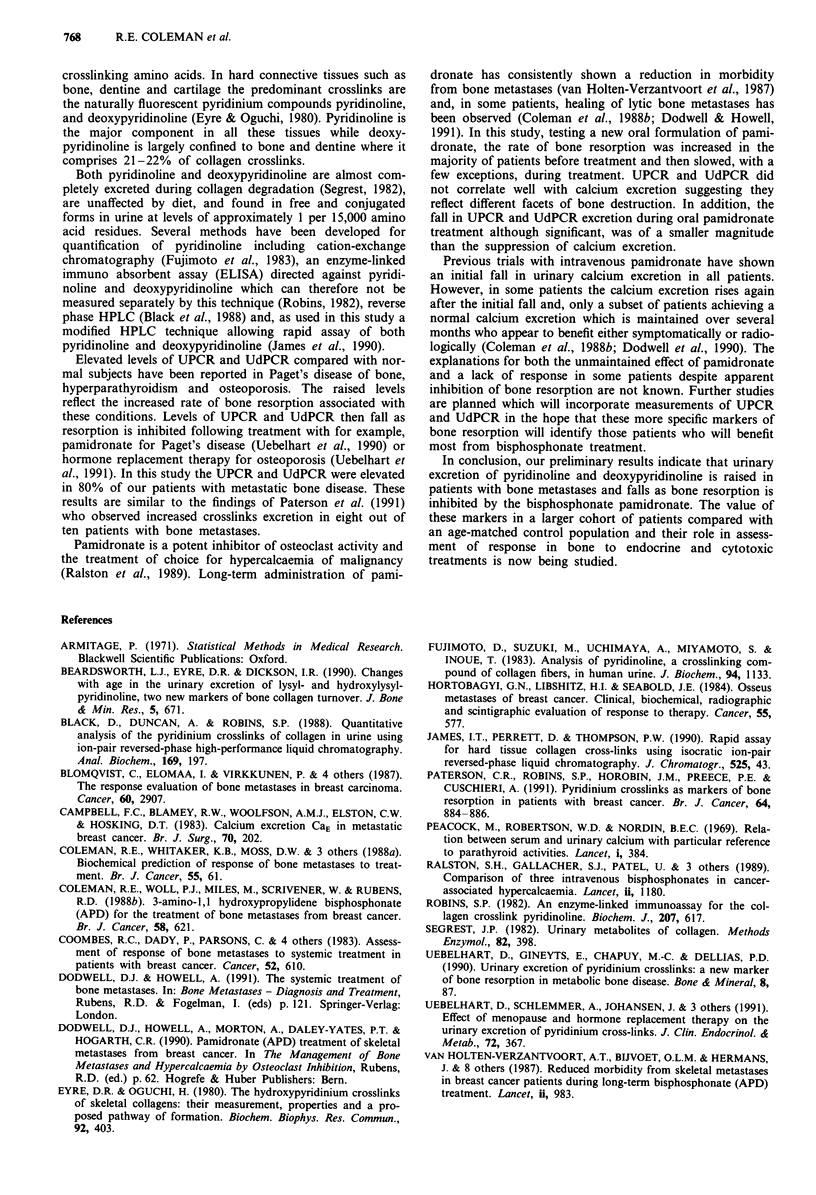


## References

[OCR_00424] Beardsworth L. J., Eyre D. R., Dickson I. R. (1990). Changes with age in the urinary excretion of lysyl- and hydroxylysylpyridinoline, two new markers of bone collagen turnover.. J Bone Miner Res.

[OCR_00430] Black D., Duncan A., Robins S. P. (1988). Quantitative analysis of the pyridinium crosslinks of collagen in urine using ion-paired reversed-phase high-performance liquid chromatography.. Anal Biochem.

[OCR_00436] Blomqvist C., Elomaa I., Virkkunen P., Porkka L., Karonen S. L., Risteli L., Risteli J. (1987). The response evaluation of bone metastases in mammary carcinoma. The value of radiology, scintigraphy, and biochemical markers of bone metabolism.. Cancer.

[OCR_00441] Campbell F. C., Blamey R. W., Woolfson A. M., Elston C. W., Hosking D. J. (1983). Calcium excretion (CaE) in metastatic breast cancer.. Br J Surg.

[OCR_00451] Coleman R. E., Woll P. J., Miles M., Scrivener W., Rubens R. D. (1988). Treatment of bone metastases from breast cancer with (3-amino-1-hydroxypropylidene)-1,1-bisphosphonate (APD).. Br J Cancer.

[OCR_00459] Coombes R. C., Dady P., Parsons C., McCready V. R., Ford H. T., Gazet J. C., Powles T. J. (1983). Assessment of response of bone metastases to systemic treatment in patients with breast cancer.. Cancer.

[OCR_00475] Eyre D. R., Oguchi H. (1980). The hydroxypyridinium crosslinks of skeletal collagens: their measurement, properties and a proposed pathway of formation.. Biochem Biophys Res Commun.

[OCR_00481] Fujimoto D., Suzuki M., Uchiyama A., Miyamoto S., Inoue T. (1983). Analysis of pyridinoline, a cross-linking compound of collagen fibers, in human urine.. J Biochem.

[OCR_00485] Hortobagyi G. N., Libshitz H. I., Seabold J. E. (1984). Osseous metastases of breast cancer. Clinical, biochemical, radiographic, and scintigraphic evaluation of response to therapy.. Cancer.

[OCR_00491] James I. T., Perrett D., Thompson P. W. (1990). Rapid assay for hard tissue collagen cross-links using isocratic ion-pair reversed-phase liquid chromatography.. J Chromatogr.

[OCR_00495] Paterson C. R., Robins S. P., Horobin J. M., Preece P. E., Cuschieri A. (1991). Pyridinium crosslinks as markers of bone resorption in patients with breast cancer.. Br J Cancer.

[OCR_00501] Peacock M., Robertson W. G., Nordin B. E. (1969). Relation between serum and urinary calcium with particular reference to parathyroid activity.. Lancet.

[OCR_00506] Ralston S. H., Gallacher S. J., Patel U., Dryburgh F. J., Fraser W. D., Cowan R. A., Boyle I. T. (1989). Comparison of three intravenous bisphosphonates in cancer-associated hypercalcaemia.. Lancet.

[OCR_00511] Robins S. P. (1982). An enzyme-linked immunoassay for the collagen cross-link pyridinoline.. Biochem J.

[OCR_00515] Segrest J. P. (1982). Urinary metabolites of collagen.. Methods Enzymol.

[OCR_00519] Uebelhart D., Gineyts E., Chapuy M. C., Delmas P. D. (1990). Urinary excretion of pyridinium crosslinks: a new marker of bone resorption in metabolic bone disease.. Bone Miner.

[OCR_00525] Uebelhart D., Schlemmer A., Johansen J. S., Gineyts E., Christiansen C., Delmas P. D. (1991). Effect of menopause and hormone replacement therapy on the urinary excretion of pyridinium cross-links.. J Clin Endocrinol Metab.

[OCR_00531] van Holten-Verzantvoort A. T., Bijvoet O. L., Cleton F. J., Hermans J., Kroon H. M., Harinck H. I., Vermey P., Elte J. W., Neijt J. P., Beex L. V. (1987). Reduced morbidity from skeletal metastases in breast cancer patients during long-term bisphosphonate (APD) treatment.. Lancet.

